# Improved and easy method for long-term *in vitro* culture of rumen ciliates *Dasytricha ruminantium*

**DOI:** 10.1016/j.mex.2024.102902

**Published:** 2024-08-10

**Authors:** Qinhui Xu, Jinying He, Yujia Wang, Jie Xiong, Weiwei Qin, Jinmei Feng

**Affiliations:** a Department of Pathogenic Biology, School of Medicine, Jianghan University, Wuhan, China; b Institute of Hydrobiology, Chinese Academy of Sciences, Wuhan, China; c Key Laboratory of Breeding Biotechnology and Sustainable Aquaculture, Chinese Academy of Sciences, Wuhan, China; d Hubei Key Laboratory of Cognitive and Affective Disorders, Jianghan University, Wuhan, China

**Keywords:** *In vitro* cultivation, Anaerobic ciliates, Rumen protozoa, *In vitro* cultivation technique of *Dasytrica ruminantium*

## Abstract

The rumen ciliates are a diverse group of protozoa residing in the rumen of ruminant animals. They are primarily found in the orders Entodiniomorphida and Vestibuliferida, playing crucial roles in the digestion and breakdown of feed within the host's rumen, closely intertwined with the host's nutrient absorption. *In vitro* monocultures of representatives of rumen ciliates are important to better study them. So far, *Entodinium caudatum* and *Epidinium caudatum*, representatives of the order Entodiniomorphida, have been successfully cultivated as a monoculture *in vitro*. However, for the order Vestibuliferida, no representative species has been established a stable monoculture *in vitro* up to date, which hampers to study their physiology and metabolism. Therefore, we have developed a simple method for the *in vitro* cultivation of *Dasytricha ruminantium*, a representative rumen ciliate in the order Vestibuliferida. Utilizing an optimized culture medium with easily obtainable components, and the cultivation process is simple. This will facilitate further research in metabolism and other studies requiring large pure live materials.

1. Filtration and separation for enriching *D. ruminantium*.
2. A culture medium (DRM) suitable for the growth of *D. ruminantium*, with easily obtainable components.
3. Simple cultivation process, facilitating the obtainment of a large number of monocultured *D. ruminantium*.

## Graphical abstract

**Figure:**
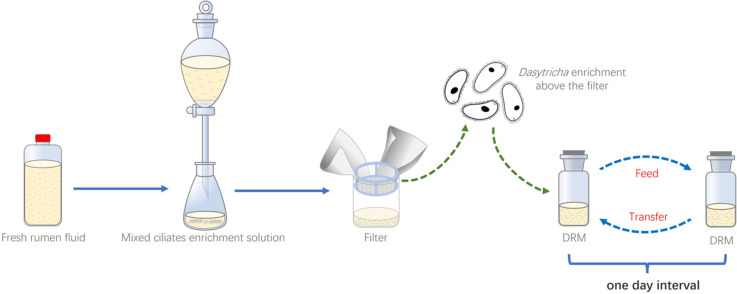

Specifications table

**Table:** 

Subject area:	Agricultural and Biological Sciences
More specific subject area:	Protozoan rumen ciliates
Name of your method:	*In vitro* cultivation technique of *Dasytrica ruminantium*
Name and reference of original method:	None
Resource availability:	None

## Background

Rumen ciliates, which are primarily classified into the order Entodiniomorphida and Vestibuliferida, comprise over 200 species [1]. They are the dominant protozoa in the rumen of ruminants, playing vital roles in stabilizing the rumen environment, degrading cellulose, preventing host toxicity and increasing methane production [2,3]. *In vitro* culture is a key foundation to study the function of rumen ciliates. It is impossible to culture any ruminal ciliate species axenically *in vitro*, and rumen ciliates are isolated from rumen fluid and cultured as monocultures or mixed cultures *in vitro* to better study them [[4], [5], [6]]. The current laboratory cultures of rumen ciliates are primarily focusing on the representatives of the order Entodiniomorphida, such as *Entodinium caudatum* and *Epidinium caudautum*, can be successfully maintained *in vitro* as monocultures [5,[7], [8], [9], [10], [11]]. For the order Vestibuliferida, only one representative, *Dasytricha ruminantium*, had been laboratory monocultured successfully for a duration of two months with a maximum population 1300 cells/ml in 60 years ago by Clarke and Hungate [4]. However, due to the complexity of the culture procedure, requirement of specific equipment, and difficulties in obtaining some dedicated culture components, such as protozoal extracts and clarified rumen fluid obtaining from the same cow which provided the rumen ciliate suspensions, the culture method of *D. ruminantium* established by Clarke and Hungate is difficult to replicate and continue. To solve these problems, we introduced an improved and simplified method using a relatively simple and available culture medium composition that supports the stable growth and proliferation of *D. ruminantium in vitro*. We successfully monocultured *D. ruminantium* using the DRM medium for up to 11 months, achieving a maximum growth density of 40,000 cells/ml.

## Method details

### Preparation and sterilization of materials

The Dasytricha ruminantium medium (DRM)

• Fetal bovine serum (VivaCell, C04001–500)
• Horse serum (DingGuo, NIS-0103)
• Autoclaved 50 × Cysteine·HCl solution
• Filter-sterilized 50 × Penicillin-Streptomycin solution
• Autoclaved 5 % sucrose (HuShi, 57–50–1) solution
• SP salt solution

Note:

1. 50 × Cysteine·HCl Solution is a 1 % aqueous solution of Cysteine·HCl (Macklin, 52–89–1).

2. 50 × Penicillin-Streptomycin Solution is a mixed aqueous solution of 3 % Penicillin G Sodium (BBI, 69–57–8) and 2.5 % Streptomycin Sulfate (BioFroxx, 3810–74–0).

3. The preparation method for SP salt solution [10] is as follows:

① Weigh 5.5 g of K_2_HPO_4_·3H_2_O, 4.0 g of KH_2_PO_4_, 0.07 g of MgSO_4_·7H_2_O, and 0.5 g of NaCl, add distilled water to make up to 800 mL, obtaining solution A.

② Weigh 0.04 g of CaCl_2_, add distilled water to make up to 100 mL, obtaining solution B.

③ Weight 6 g of NaHCO_3_, add distilled water to make up to 100 mL, obtaining solution C.

④ Sterilize solutions A, B, and C separately with 121 °C for 15 min, then mix them and gas with CO_2_ for 5 min to obtain SP salt solution, seal for later use.

The components of DRM are as follows (v/v): 5 % fetal bovine serum (FBS), 5 % horse serum (HS), 2 % Cysteine·HCl solution, 2 % Penicillin-Streptomycin solution, 3 % sucrose solution and 83 % SP salt solution. According to the required amount of culture medium, mix an appropriate amount of the above solutions and place them in a 39 °C preheated environment for later use.

### Isolation and enrichment of *D. ruminantium*

**Fig. 1:**
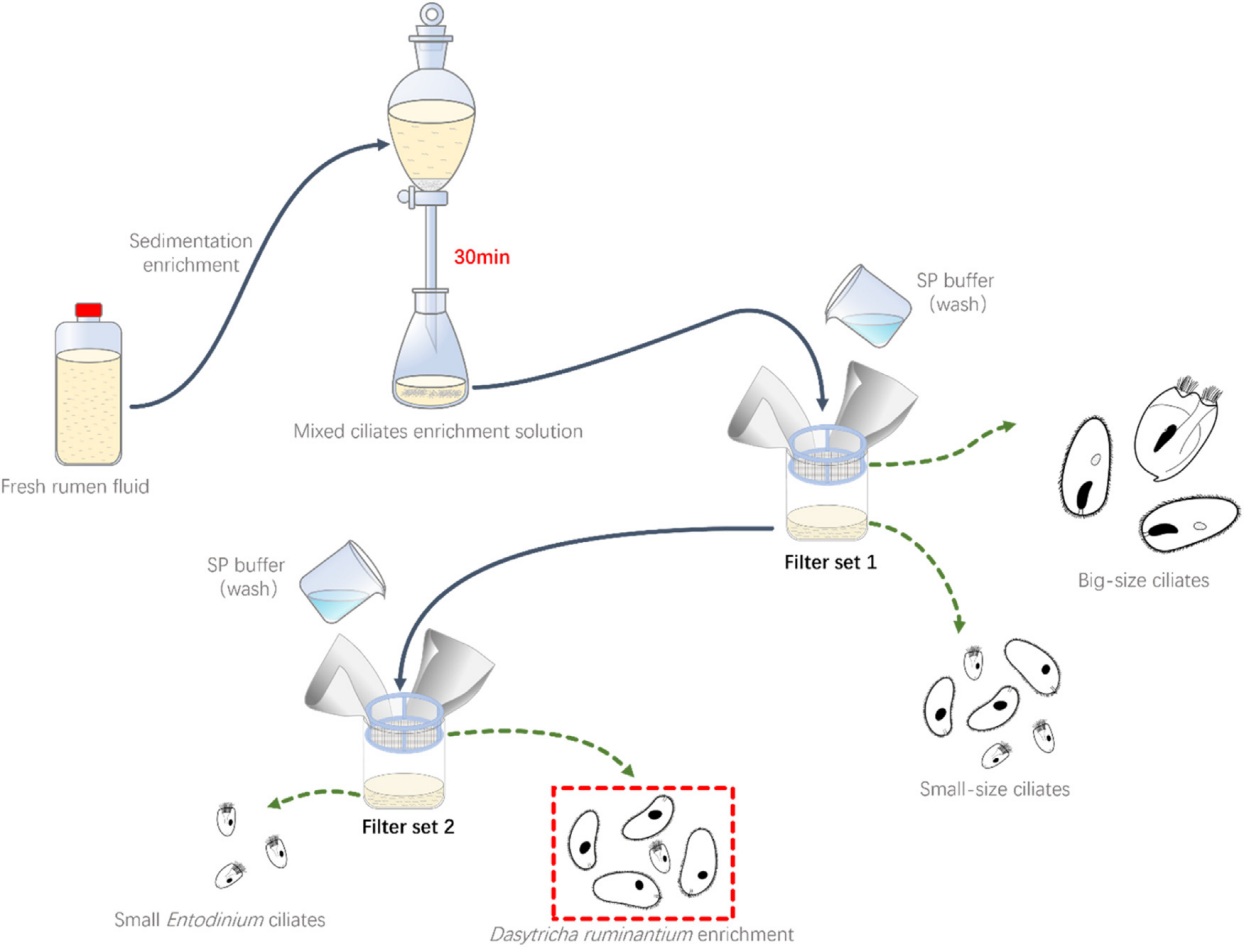
Schematic of isolating and enriching of *D. ruminantium*.

Using the sterilized rumen fluid collection cannula to collect fresh rumen fluid, then filter the rumen fluid using four-layer gauze, seal and promptly send it to the laboratory at 39 °C. Transfer the collected rumen fluid to a separation funnel and gas with CO_2_ for 3 - 5 min, then stand it in a 39 °C incubator for 30 - 60 min. Collect the rumen ciliates that have settled to the bottom of the funnel into a small beaker containing SP salt solution, obtaining a mixed rumen ciliates enrichment suspension. Subsequently, filter the mixed rumen ciliates enrichment suspension through 50 µm and 25 µm nylon mesh sequentially, collect the retained ciliates from 25 µm nylon mesh with SP salt solution, obtaining the enriched suspension of *D. ruminantium*, use this enriched *D. ruminantium* suspension as an inoculum, which the proportion of *D. ruminantium* is approximate 70–90 %, gas with CO_2_ and keep it warm at 39 °C for later use (Fig. 1).

Note:

1. After inoculation of *D. ruminantium* into DRM, there may be a reduction in the short term, so the initial inoculation quantity should not be too low. It is recommended that the initial inoculation quantity is between 500 - 1000 cells/ml.
2. The *D. ruminantium* obtained on a 25 µm nylon mesh may contain a few other species of rumen ciliates, but they will gradually disappear during the cultivation process due to an unfavorable culture medium and culture condition.
3. The optimal time to collect the rumen ciliates from the separation funnel is when a significant amount of white sediment (*i.e.*, rumen ciliate enrichment) has formed at the bottom of the separation funnel, and plant residues have floated to the top layer of the liquid. However, the condition of rumen fluid samples collected from different individual hosts and at different times may vary, resulting in different optimal separation durations. According to our long-term experimental observations, rumen ciliates can be effectively separated within 30 - 60 min for different samples.

### Method and procedure

Add 8 ml of preheated DRM and 2 ml of inoculum to a 25 ml anaerobic culture bottle, inject CO_2_ to the top of the bottle for 5 – 10 s, then quickly seal the bottle cap and place it in a 39 °C shaking incubator at 150 rpm for cultivation. After 24 h of cultivation, add 300 µl of 5 % sucrose solution to the anaerobic culture bottle. After 48 h, pause the shaking incubator and let it stand for 30 - 60 min to allow *D. ruminantium* to settle to the bottom of the culture bottle, replace 50 % of the original culture medium with fresh medium, inject CO_2_ for 5 – 10 s and seal the bottle cap, return it to the shaking incubator for continued cultivation. Repeat this cycle to ensure continuous growth and proliferation of *D. ruminantium*.

**Fig. 2:**
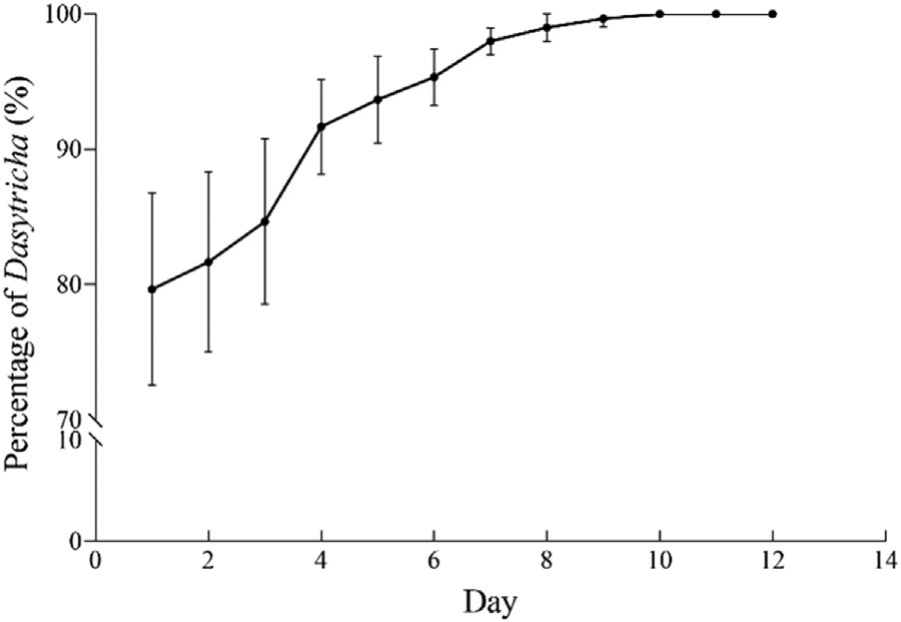
The proportion change of *D. ruminantium* in the culture.

**Fig. 3:**
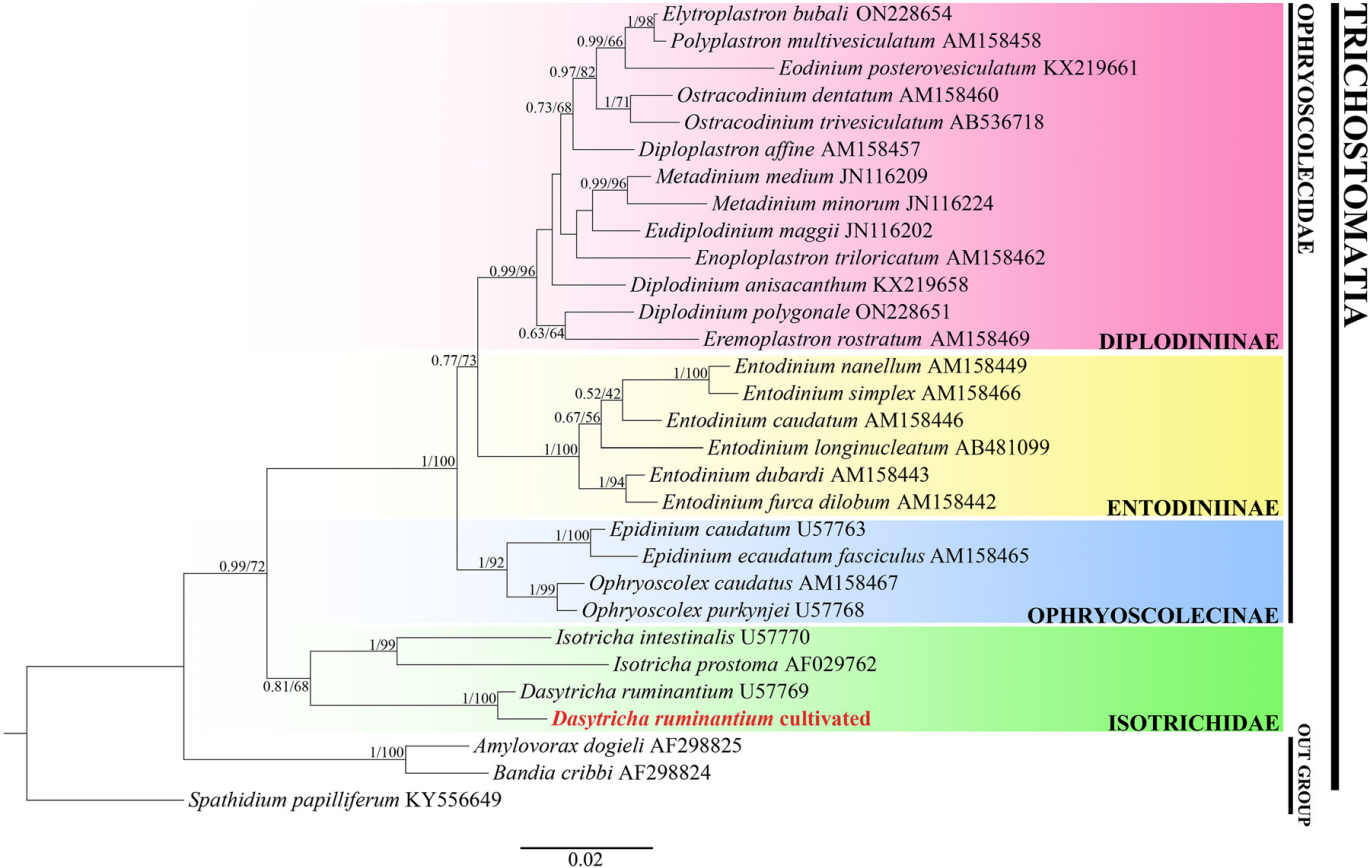
Phylogenetic analysis of rumen ciliates based on 18S rRNA gene sequences. ML analysis was performed using IQ-tree software, and BI analysis was conducted using the MrBayes software with the GTR+F + I + G4 model. New sequence obtained in this study is highlighted in bold red. The numbers at nodes represent BI posterior probabilities and ML bootstrap support (%) values. The scale bar corresponds to 0.02 expected substitutions per site.

After one to two weeks of cultivation, the small number of *Entodinium* from the inoculum disappeared from the culture due to unsuitable cultivation conditions. At this point, microscopic observation of the culture detected only *D. ruminantium* (Fig. 2). Further, three samples, each containing 50 cultured rumen ciliates, were randomly collected from the culture and washed three times by sterile SP salt solution. Subsequently, the 18S rRNA gene was amplified using the REDExtract-N-Amp™ Tissue PCR kit (Sigma Aldrich, XNAT-10RXN) using ciliate-specific primers P.324f (5’-CGGTAGTGTATTGGACWACCATG-3’) and P.1747r_2 (5’-CTCTARGTGATRWGRTTTAC-3’) reported previously [12]. The amplified and purified 18S rRNA gene products were sequenced and analyzed for taxonomical classification of the cultured rumen ciliates. The sequencing results showed no noise peak in all three samples, and the obtained 18S rRNA gene of the three samples is the same. Using the obtained 18S rRNA gene sequence as query to blast search against the GenBank database, the best hit is the 18S rRNA gene of *D. ruminantium* (Gene ID: U57769), with an E-value of 0 and a percent identity of 99.51 %. Furthermore, the sequenced 18S rRNA gene of the cultivated rumen ciliate was named *D. ruminantium* cultivated, integrated with 26 rumen ciliate sequences and 3 outgroup sequences from GenBank, were subjected to phylogenetic analysis using bayesian inference (BI) and maximum likelihood (ML) methods through PhyloSuite v1.2.3 and its plugins [13]. The topologies of the BI tree and the ML tree are consistent (Fig. 3), and the 18S rRNA gene sequence of *Dasytricha ruminantium* cultivated in this study is sister grouped with the reported 18S rRNA gene sequence of *D. ruminantium* in GenBank with full support (1.0 BI/100 % ML). These results indicate that the cultured ciliates in this study is *D. ruminantium*, and a stable laboratory monoculture of *D. ruminantium* was established.

### Method validation

**Fig. 4:**
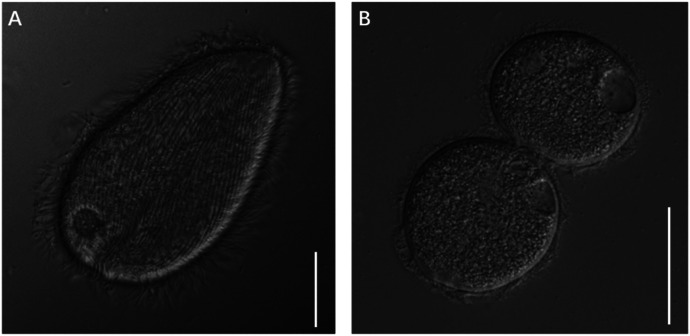
Morphology of *D. ruminantium* from *in vitro* culture. (A) *D. ruminantium* after 30 days of *in vitro* cultivation. (B) *D. ruminantium* in the process of division. Scale-bars: A 20 µm; B 50 µm.

**Fig. 5:**
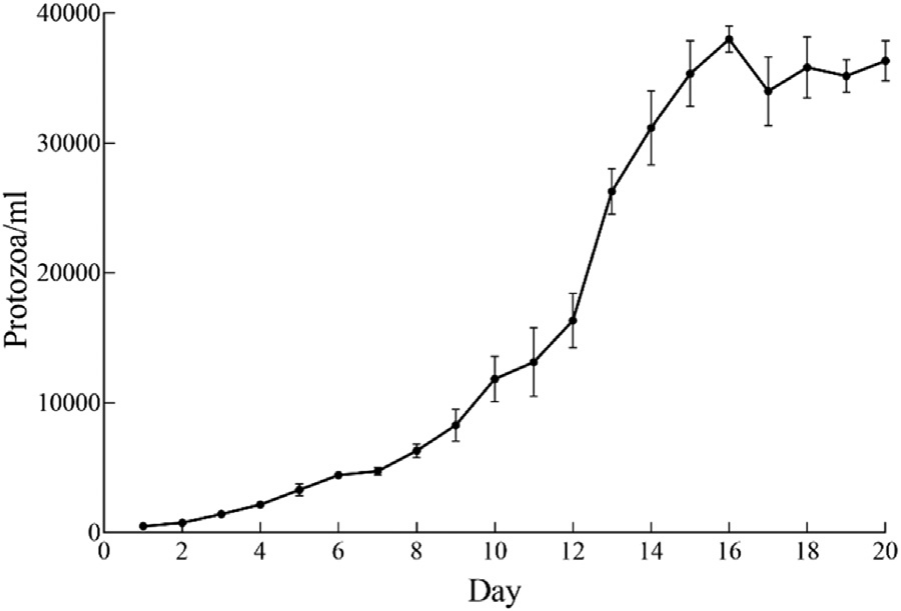
The growth curve of the *in vitro* cultured *D. ruminantium*.

Through the above methods, we have successfully maintained *D. ruminantium* in a monoculture for >11 months, with vigorous growth and proliferation observed (Fig. 4). With an initial inoculum density of 500 cells/ml of *D. ruminantium*, the growth density reached 38,000 cells/ml after 16 days of cultivation (Fig. 5).

**Fig. 6:**
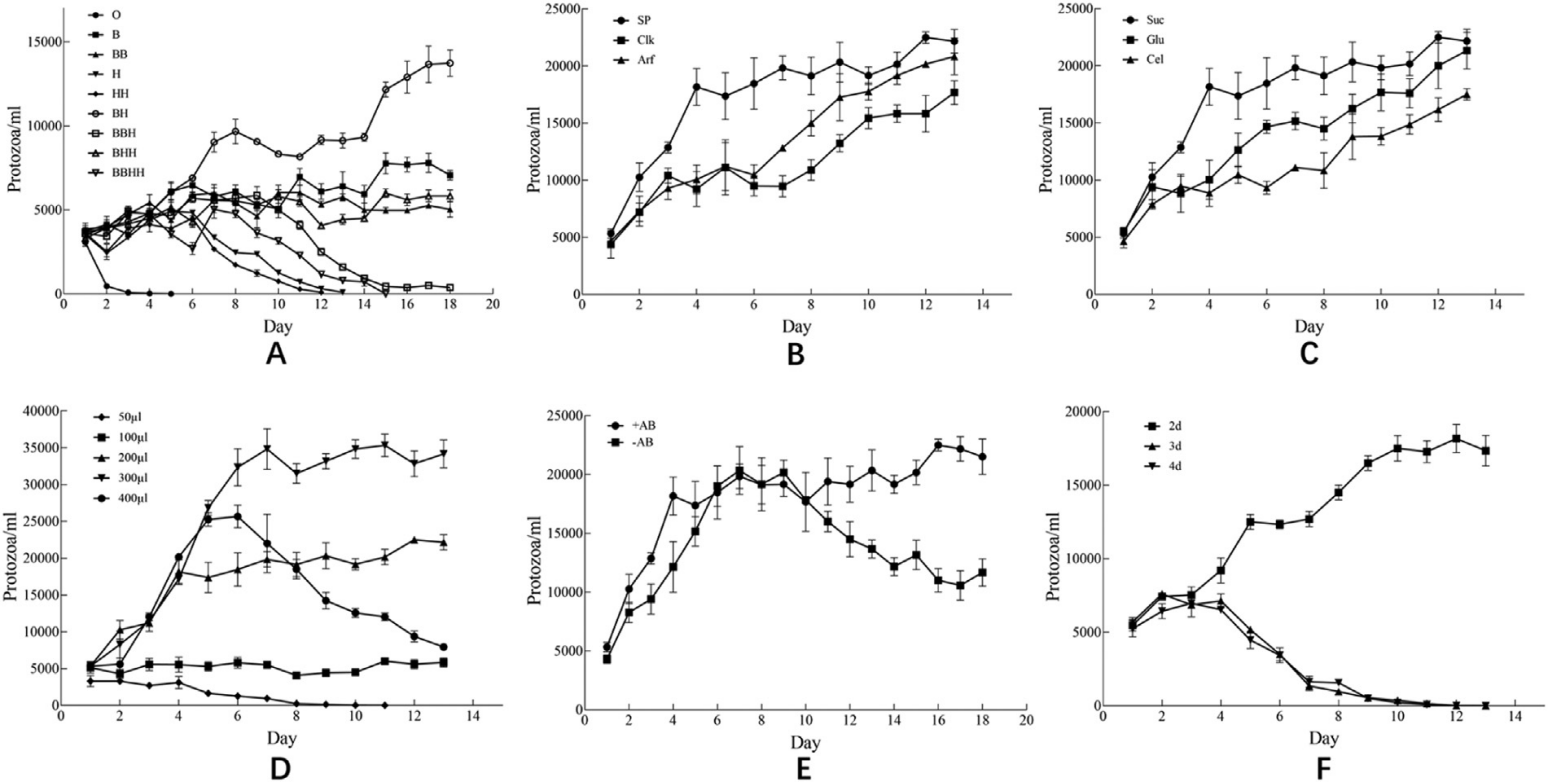
Growth curves of *D. ruminantium* under different conditions. (A) The impact of different types and concentrations of serums on the growth of ciliates. O: no serum; B: fetal bovine serum (FBS); H: horse serum (HS). Each "B" and "H" represents the addition of the respective serum at a concentration of 5 % by volume. (B) SP: SP salt solution; Clk: salt solution referenced to Clarke and Hungate's method; Arf: Artificial rumen fluid. (C) Suc: sucrose; Glu: glucose; Cel: cellobiose. (D) Daily volume of sucrose solution added. (E) +AB: culture added with antibiotics; -AB: culture without antibiotics; (F) Frequency of medium replacement.

Due to the difficulties in obtaining protozoal extracts and the clarified rumen fluid obtaining form the same cow which provided the ciliate suspensions in Clarke and Hungate method, we improved to use the commercially obtained serums to culture *D. ruminantium*. Initially, we attempted to rely solely on FBS, however, the outcomes proved to be unsatisfactory. Referring to the method of culturing *Balantidium ctenopharyngodoni* [14], we tested the cultivation effectiveness of *D. ruminantium* by using different serums. We found that combining 5 % FBS with 5 % HS is the best condition to enhance the cultivation effectiveness of *D. ruminantium* (Fig. 6A). After comparing the effects of SP [10], Clk [4], and Arf [8] salt solutions on the cultivation of *D. ruminantium*, we found that under the same conditions for other variables, the SP salt solutions yielded the best cultivation results (Fig. 6B). As to the substrate, we tested sucrose, glucose, and cellobiose, and the results indicated that sucrose was the most effective one with a concentration of 0.15 %, and the highest density of cultured *D. ruminantium* is over 40,000 cells/ml (Fig. 6C-D). Antibiotics and antioxidants are not essential, but their addition is beneficial to limit the growth of bacteria and reduce the redox potential of the culture system to promote the growth of *D. ruminantium* (Fig. 6E).

After comprehensive consideration of various testing conditions, we have defined the optimal medium formulation, which includes 5 % FBS, 5 % HS, 2 % Cysteine·HCl solution, 2 % Penicillin-Streptomycin solution, 3 % sucrose solution and 83 % SP salt solution.

Furthermore, we tested the cultivation effectiveness of *D. ruminantium* with different medium change frequencies. The results indicated that refreshing the medium every two days yielded the best effect to improve the growth of *D. ruminantium* (Fig. 6F). Therefore, the final cultivation protocol involves feeding substrate daily and change medium every two days. During the laboratory monoculture of *D. ruminantium*, gases such as CO_2_, H_2_, and CH_4_ are produced due to the metabolisms of bacteria and *D. ruminantium* in the medium [15]. Therefore, if the bottle cap is opened directly during a medium change, the dissolved gases in the medium will be released, resulting in the *D. ruminantium* at the bottom to float, which could lead to an increased loss of cultured ciliates along with the discarded old medium. Therefore, before changing the medium, it is advisable to vent the culture bottle by making punctures with a syringe needle, allowing it to stand for some time before the medium change, which helps to minimize *D. ruminantium* losses.

## Limitations

None.

## Ethics statements

Animals used in the present study were treated under the guidelines of the Regulations for the Administration of Affairs Concerning Experimental Animals (Ministry of Science and Technology, China, 2013) with the approval number JHDXLL2020–002.

## N material *and/or* additional information [OPTIONAL]

None.

## CRediT authorship contribution statement

**Qinhui Xu:** Conceptualization, Methodology, Investigation, Formal analysis, Visualization, Writing – original draft, Writing – review & editing. **Jinying He:** Methodology, Investigation, Writing – original draft. **Yujia Wang:** Methodology, Investigation, Writing – original draft. **Jie Xiong:** Formal analysis, Visualization, Writing – review & editing. **Weiwei Qin:** Visualization, Writing – review & editing. **Jinmei Feng:** Conceptualization, Methodology, Investigation, Formal analysis, Visualization, Writing – original draft, Writing – review & editing, Supervision, Funding acquisition.

## Declaration of competing interest

The authors declare that they have no known competing financial interests or personal relationships that could have appeared to influence the work reported in this paper.

## Data Availability

Data will be made available on request.
